# IMACEL: A cloud-based bioimage analysis platform for morphological analysis and image classification

**DOI:** 10.1371/journal.pone.0212619

**Published:** 2019-02-22

**Authors:** Yuki Shimahara, Ko Sugawara, Kei H. Kojo, Hiroki Kawai, Yuya Yoshida, Seiichiro Hasezawa, Natsumaro Kutsuna

**Affiliations:** 1 Department of Integrated Biosciences, Graduate School of Frontier Sciences, University of Tokyo, Kashiwanoha, Kashiwa, Chiba, Japan; 2 Research and Development Division, LPixel Inc., Chiyoda-ku, Tokyo, Japan; 3 Graduate School of Science and Technology, Sophia University, Chiyoda-ku, Tokyo, Japan; University of California Riverside, UNITED STATES

## Abstract

Automated quantitative image analysis is essential for all fields of life science research. Although several software programs and algorithms have been developed for bioimage processing, an advanced knowledge of image processing techniques and high-performance computing resources are required to use them. Hence, we developed a cloud-based image analysis platform called IMACEL, which comprises morphological analysis and machine learning-based image classification. The unique click-based user interface of IMACEL’s morphological analysis platform enables researchers with limited resources to evaluate particles rapidly and quantitatively without prior knowledge of image processing. Because all the image processing and machine learning algorithms are performed on high-performance virtual machines, users can access the same analytical environment from anywhere. A validation study of the morphological analysis and image classification of IMACEL was performed. The results indicate that this platform is an accessible and potentially powerful tool for the quantitative evaluation of bioimages that will lower the barriers to life science research.

## Introduction

Recent developments in microscopic and image processing technologies have led to new findings in the life sciences. With the evolution of imaging devices, such as microscopes, MRI, and CT, image data in the life sciences are increasingly detailed. In particular, the development of visualisation techniques, such as the use of fluorescence microscopy and fluorescent probes, facilitate the analysis of biological structures and diversify molecular imaging. Therefore, it is becoming critical to analyse these bioimage data efficiently and quickly in quantitative studies [1,2]. Generally, the analysis of large and detailed images is very laborious and time-consuming, and is a burden for researchers. In addition to advances in imaging devices, a variety of open source and commercial image analysis software (e.g., ImageJ [3], ImagePro, and Photoshop) and libraries for programming languages (e.g., OpenCV and Bioconductor) have been developed; however, their use requires specialist knowledge.

Machine learning is also used to analyse large quantities of bioimage data. Using this technique, it has become possible to automate or semi-automate analysis for the target extraction and classification of diverse and massive numbers of biological images [4,5]. Deep learning-based convolutional neural networks are expected to be useful for single-cell experiments with high-throughput and high-content screening [6,7]. A report on using nonlinear dimensionality reduction in combination with deep learning to reconstruct cell cycle and disease progression has demonstrated the efficiency of applying machine learning techniques to objective biological prediction [8]. For instance, we previously proposed a system that combines machine learning and active learning [9] for subcellular localisation, mitotic phase classification, and the discrimination of apoptosis in images of plant and human cells. This system achieved an accuracy level greater than or equal to that of the annotators [10].

Although advanced image processing and machine learning techniques are necessary in life science studies, many research labs are ill-equipped to perform bioimage analysis that uses advanced imaging technologies and many computing resources. For generic morphological analysis, such as counting a number, measuring an area, and extracting several features of a shape, researchers need information about the signal/background setting, noise reduction filtering, binaiysation setting, and particle analyser function in de facto-standard image processing software ImageJ, and must manually choose particular algorithms for each specific research purpose and tune the parameters manually. Additionally, for classification analysis, almost all software and analytical environments require skills for programing languages to input commands. Hence, although image processing plays an important role in quantitative data analysis for life sciences, the current available image processing solutions are too complicated for most researchers to use. Thus, user-friendly software for image analysis is needed to expand the use of imaging technologies throughout the life sciences.

IMACEL is a cloud-based image analysis platform developed for automatic classification and morphological analysis. Because all image processing and machine learning are performed by virtual machines in the cloud, it is not necessary to set up powerful laboratory computers or workstations. IMACEL’s target data includes various types of microscopic bioimages. The most important feature in IMACEL is the new user interface for researchers with limited knowledge of image processing. IMACEL suggests multiple candidates for morphological analysis, allows users to select the most efficiently processed images (S1 Movie). This allows users to determine appropriate procedures quickly and easily. In addition to morphological analysis, IMACEL can perform automatic image classification from uploaded annotated images using random forests and a deep learning algorithm.

The contributions of this study are as follows:

- We present a tool that enables life science researchers with limited image processing experience and computing resources to automatically and quantitatively analyse microscopic image data.
- We verify the morphological analysis of the system by evaluating the number and size of stress granules in images using the batch process function. Moreover, we evaluate the classification analysis of cell cycle progression using machine learning techniques on the IMACEL platform.

The adoption of IMACEL in life science research has the potential to improve the quality and quantity of research, particularly for researchers who would not otherwise have the experience and resources to perform such investigations.

## Results

**Fig 1:**
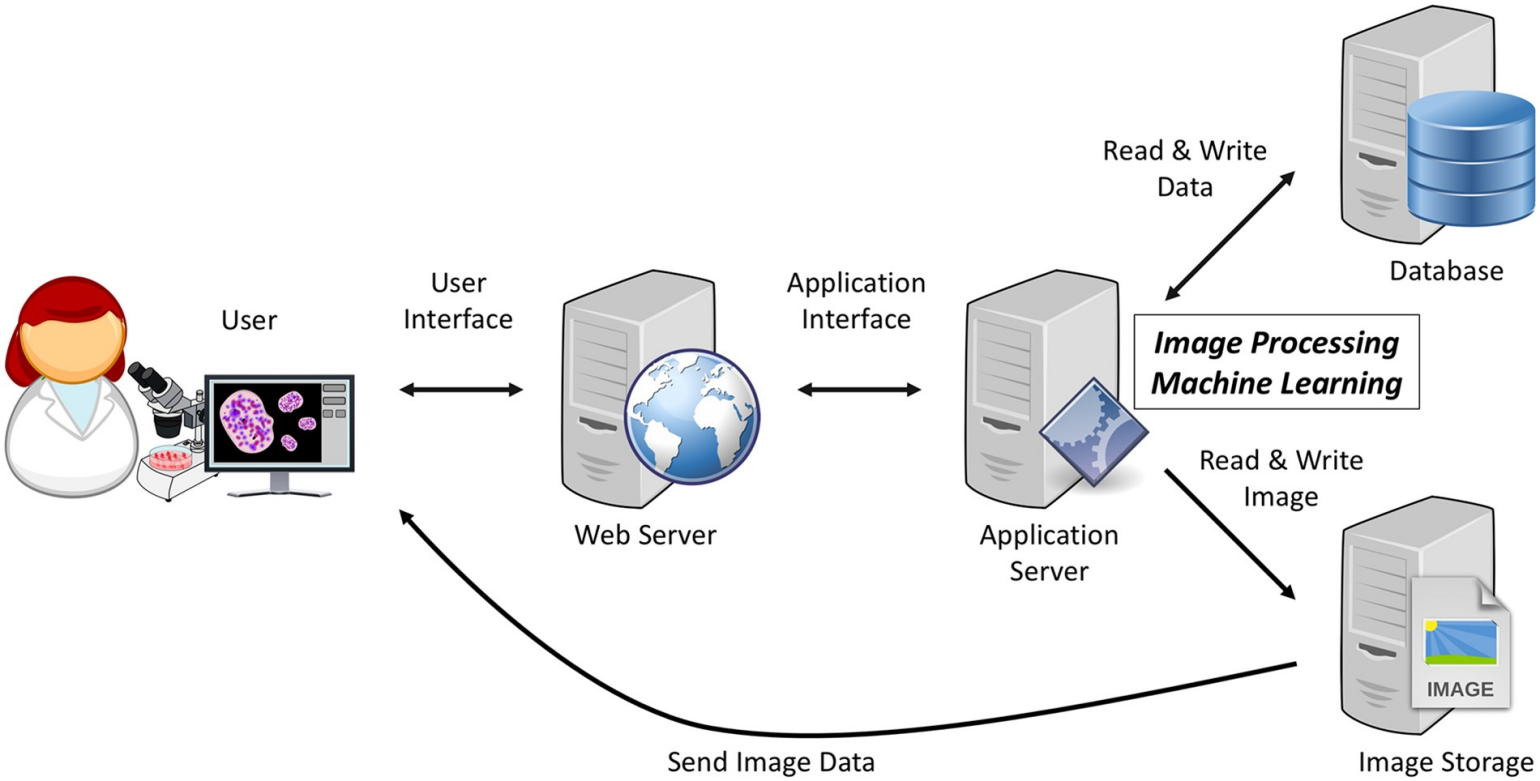
Architecture of IMACEL, a cloud-based image processing and machine learning platform for life science researchers. The entire process of image processing is performed in the cloud using high-performance virtual machines. The public-domain images used in this figure were obtained from Openclipart.

As illustrated in Fig 1, IMACEL is a cloud-based image processing platform. Researchers upload images to the web server through a web browser. Image processing and image classification are performed by high-performance virtual machines, and the processed image data are sent back through the web browser. IMACEL has the following two independent functions: a particle analyser for morphological analysis and a classifier for bioimage classification.

### Validation of the IMACEL particle analyser

**Fig 2:**
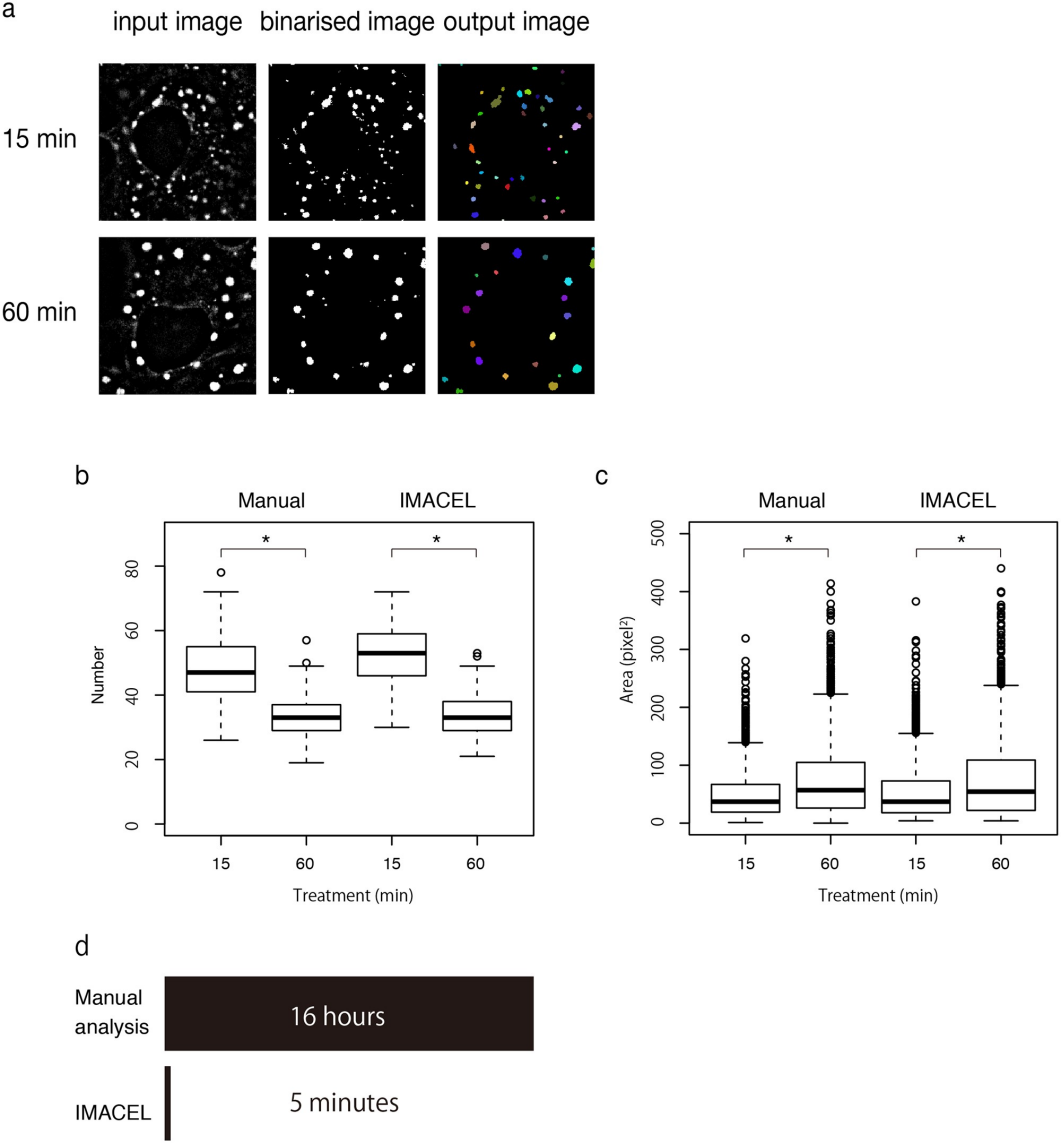
Results of the IMACEL particle analyser for extracting and evaluating stress granules in COS7 cells. (a) Input image, binarised image, and output image of the IMACEL particle analyser. Comparison of the distribution of the number (b) and size (c) of stress granules against stress treating time evaluated using manual evaluation and IMACEL. Asterisks indicate significant differences (Mann–Whitney U test) between cells treated with 0.5 μM sodium arsenite for 15 min and 60 min (in number: *p* = 2.568 × 10^−13^ and *p* < 2.2 × 10^−16^, in size: *p* < 2.2 × 10^−16^ and *p* < 2.2 × 10^−16^). (d) Total time spent on manual analysis versus the computational time of the IMACEL particle analyser. We measured 65 images each for specimen treated for 15 min and 60 min.

We validated the morphological analysis of the IMACEL particle analyser by determining how similar its extracted features were to those of a manual evaluation. We focused on immunologically labelled stress granules because the shapes of the organelles are oval and traced easily by manual evaluation (as shown in S1 Fig). It has been reported that a treatment of sodium arsenite induces the development of stress granules in a time-dependent manner [11–13]. Therefore, COS7 cells treated with 0.5 μM sodium arsenite for 15 min and 60 min were analysed with respect to the size and number of stress granules formed during treatment. We confirmed that the stress granules were segmented appropriately by the IMACEL particle analyser (Fig 2a). As expected, there were significant differences in the number and size of stress granules between the 15 min and 60 min treatments, and the morphological analysis of IMACEL yielded results that were very similar to those of the manual evaluation (Fig 2b and 2c). The batch process of the IMACEL particle analyser (65 images each for specimen treated for 15 min and 60 min) was finished in approximately 5 min. By contrast, manual evaluation by tracing each stress granule took approximately 16 h (Fig 2d, S2 Movie). These results indicate that the IMACEL particle analyser can evaluate the morphology of particles quantitatively and rapidly, with high accuracy.

### Validation of the IMACEL classifier

**Fig 3:**
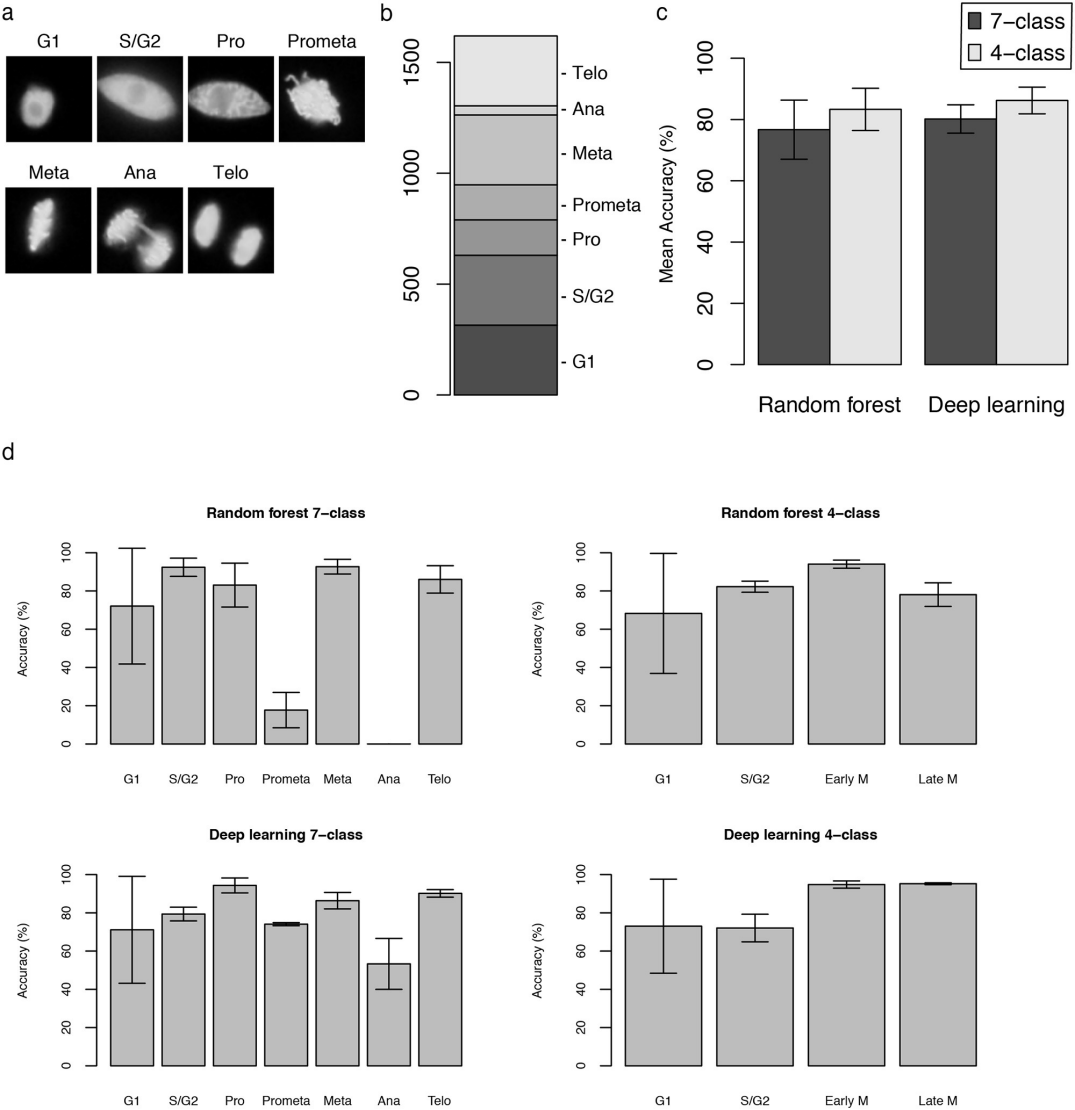
Results of the IMACEL classifier for cell cycle classification with nucleuses visualised using fluorescent images. (a) Representative images of each cell cycle in suspension-cultured plant cells. Nuclear regions were visualised using RFP-Histone H2B. (b) Distribution of the number of dataset images in each class. (c) Mean accuracy of cell cycle classification in seven-class and four-class classification using random forests and deep learning. For four-class classification, the prophase, prometaphase, and metaphase were integrated into the early mitotic phase. Anaphase and telophase were integrated into the late mitotic phase. (d) Accuracy of each cell cycle classification with bars representing the standard deviation based on three independent experiments.

To validate the IMACEL classifier, a classification of cell cycle progression in tobacco BY-2 suspension-cultured cells was performed using two machine learning methods: random forests and deep learning. Because of its highly synchronised cell cycle progression [14], this cell cycle is very suitable for bioimage classification (Fig 3a). Moreover, synchronised BY-2 cells are one of the most suitable suspension-cultured cells for observing each cell cycle. Nucleuses and chromosomes were visualised using histone H2B-RFP [14,15], and the image features were extracted using the LPX296 feature extractor (formerly the KBI feature extractor [10]) and a higher-order local autocorrelation feature extractor.

The classification dataset was composed of 1,619 images of seven classes (Fig 3b). To avoid overfitting, the mean accuracy was calculated using three-fold cross-validation. The user interface of the actual IMACEL classifier was shown in S3 Movie.

The random forest IMACEL classifier [10] identified seven cell cycle classes with a mean accuracy of approximately 76.69% and four classes with a mean accuracy of approximately 83.31% (Fig 3c). S/G2 and metaphase were classified with high accuracy, but prometaphase and anaphase were classified with comparatively low accuracy (Fig 3d).

By contrast, the deep learning method in IMACEL managed to identify seven cell cycle classes with a mean accuracy of approximately 80.17% and four classes with a mean accuracy of approximately 86.21% (Fig 3c). The mean accuracies of prometaphase and anaphase classification increased when deep learning classification was used (Fig 3d).

These results indicate that IMACEL can automatically classify images without requiring researchers to have advanced knowledge of various image processing and machine learning techniques.

## Methods

### Implementation and architecture of the IMACEL platform

IMACEL is a cloud-based image processing platform that runs on Windows, Mac OS X, and Linux. The image processing core modules of IMACEL were developed using Python 3 and OpenCV, and computation is performed on a virtual machine using the Microsoft Azure service.

A virtual machine with the standard D2 v2 instance type (2 vCPU, 7 GB RAM) was used in this study. Azure Storage was used as the image storage server. To connect to the storage server from a web application server, the Azure Storage SDK for Python was used. The database and web server used URLs for their connections to the storage server.

### Security of the IMACEL platform

IMACEL used SSL/TLS to establish a secure connection between the web browser, web server, application server, and storage server. To grant limited access to resources in the storage server, a shared access signatures (SAS) provided by Azure Storage was used.

### Cloud-based image processing

To use IMACEL, researchers upload images to the web server through a web browser, and the images are processed by high-performance virtual machines running on the Microsoft Azure platform that are able to communicate with the system’s database. Processed image data are sent back to the researchers through the web browser. The maximum data size for uploading images depends on the type of web browser. For example, Internet Explorer 11 has a limitation of 4 GB for file uploading.

### Interface of IMACEL with a click-based user interface

**Fig 4:**
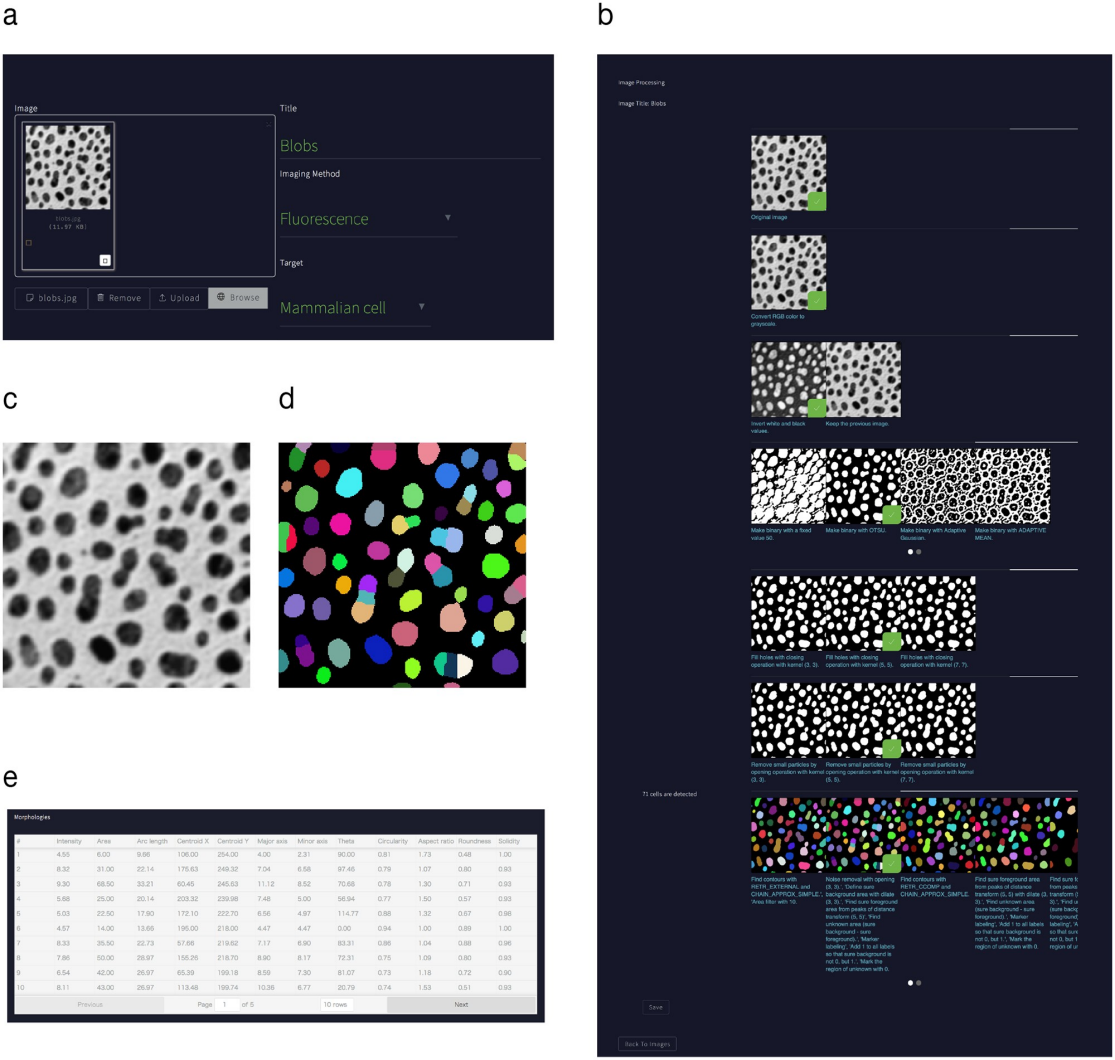
Interface of the IMACEL particle analyser. (a) The image title, imaging method, and specimen type must be provided to begin each image processing procedure. (b) Click-based user interface of the IMACEL particle analyser. Users click on the image to select the most suitable processed image for each procedure, such as noise reduction, binarisation, and postprocessing. (c) Input image, (d) segmentation output image, and (e) quantitative segmentation output.

The IMACEL platform includes a novel click-based interface designed for researchers who have no advanced image processing knowledge (Fig 4). Researchers can upload images to IMACEL, specifying the imaging method (e.g., fluorescence, bright field, or electron microscopy) and imaging target (e.g., bacteria, yeast, mammalian cells, or brain tissue) to enable the IMACEL particle analyser to provide practical suggestions (Fig 4a). In the image processing procedure, users click on the most appropriate processed image shown in the browser (Fig 4b). This clickable user interface allows researchers at all skill levels to extract particles quantitatively and objectively from raw input images (Fig 4c and 4d).

Several watershed algorithms are available at the last stage of the procedure. Additionally, several morphological features of particles, such as a number, area, roundness, fitted ellipse long and short axes, centroid coordinates, and solidity, are extracted automatically.

The IMACEL platform is designed for scientific image processing with a focus on bioimaging. Hence, all suggested procedures are appropriate for maintaining bioimage integrity (S1 Table). To enable the archiving of image processing procedures in each researche’s experimental notes, an image processing report is also provided (Fig 4e, S4 Movie).

### Classification algorithms in the IMACEL classifier

Two classification algorithms are implemented in the current version of IMACEL: a random forest and a deep learning algorithm. The convolutional neural network architecture of AlexNet, which was first place in the ImageNet Large Scale Visual Recognition Challenge 2012 (ILSVRC2012) [16], is used. AlexNet consists of eight layers: five convolutional layers and three fully connected layers. Moreover, the version used in IMACEL was pretrained on the data used in ILSVRC2012.

### Cell culture of mammalian and plant cells

The tobacco (*Nicotiana tabacum*) BY-2 cell line was diluted 95-fold with a modified Linsmaier and Skoog medium supplemented with 2,4-D at weekly intervals, as previously described [17]. The cells were agitated on a rotary shaker at 130 rpm at 27 °C in the dark. The cell cycle progression was synchronised with 5 mg^−1^ aphidicolin (Sigma), as previously described [17]. A transgenic BY-2 cell line, stably expressing an RFP-Histone H2B fusion protein, could be maintained and synchronised by procedures similar to those used for the original BY-2 cell line.

African green monkey kidney fibroblast-derived COS7 cells were obtained from the RIKEN BioResource Center and cultured in high glucose Dulbecco’s modified Eagle’s medium (Gibco) supplemented with 10% qualified heat inactivated fetal bovine serum from USDA-approved regions (Gibco), 50 U/mL penicillin-50 μg/mL streptomycin (Gibco), 2 mM L-glutamine (Gibco), 1 mM sodium pyruvate (Gibco), MEM nonessential amino acids (Gibco), and 55 μM 2-mercaptoethanol (Gibco) at 37 °C in 5% CO_2_.

### Stress treatment and immunofluorescence labelling

COS7 cells cultured onto a 35-mm glass-based dish (IWAKI) were treated with 0.5 μM sodium arsenite (Fluka) for 15 min or 60 min and fixed with 3% paraformaldehyde (Sigma Aldrich) and 0.1% glutaraldehyde (Sigma Aldrich) at 37°C in 5% CO_2_ for 10 min. COS7 cells were permeabilised with 0.2% Triton-X 100 (SIGMA) and blocked at 37°C in 5% CO_2_ for 30 min with 10% goat serum (Life Technologies) and then incubated for 30 min with primary antibodies, rabbit polyclonal anti-PABP antibody (Abcam), diluted in Can Get Signal Solution A (TOYOBO). After washing with 0.2% Triton-X 100 and PBS, the cells were incubated with Alexa 488 labelled goat anti-rabbit secondary antibodies diluted in Can Get Signal Solution A.

### Microscope

For the observation of the cell cycle in BY-2 cells, the cells were imaged using fluorescent microscopy (FSX100, Olympus, Tokyo, Japan). To extract the nuclear regions, the images were processed manually using ImageJ.

To observe the COS7 cells, they were imaged using fluorescence microscopy (N-STORM, Nikon, Tokyo, Japan). Noise in the fluorescent microscopy image was reduced with a difference of Gaussian filter using ImageJ.

### Manual evaluation of the number and size

To evaluate the number and size of stress granules, boundaries were traced manually using ImageJ software. Manual evaluations were performed by two researchers who were not involved in this study to avoid biases that could overestimate the differences in treatment effect and underestimate the differences between the results of the manual evaluation and IMACEL particle analyser.

Four authors of the current paper are experts in plant cell division. Hence, they annotated the training data for the classification of cell cycle progression in tobacco BY-2 suspension-cultured cells.

### Statistical information

To evaluate the differences in the number of stress granules, the Mann–Whitney U test was calculated using free statistical software R and RStudio versions 3.3.1 and 1.1.383, respectively.

## Discussion

The development of the IMACEL platform was based on two design concepts. The first concept is that of a novel clickable-based user interface. Existing image processing software, such as ImageJ or Photoshop CC, requires researchers to actively select the desired function from a list of image processing procedures. Because there is so much flexibility in the function selection, mistakes can be made if inappropriate image processing procedures are used. For example, a nonlocal mean filter [18], which is an effective noise reduction method, performs smoothing using similar intensity distributions from distant regions independently of whether the regions are biologically identical or not. Therefore, when such filtering is implemented in image processing software, researchers should avoid using it. By contrast, the IMACEL particle analyser effectively restricts the functions that can be selected by those unfamiliar with image processing. Additionally, batch processing is easily performed without the need to write macro functions in a programming language.

The second concept is that of a cloud-based image processing platform. Generally, machine learning requires extensive computing resources. The construction of an analytical environment is too complex for many biological researchers. Moreover, high-performance machines are expensive to establish in each laboratory. In IMACEL, because image processing and machine learning are performed on high-performance virtual machines, users can freely access their own analytical environment via a web browser from anywhere. Additionally, because IMACEL stores previous analytical images, the platform could play the role of an image management tool.

We developed this platform for researchers in the broad field of life sciences. Microscopic images are more often observed than MRI images in some life science journals. Therefore, we focused this validation study on (fluorescence) microscopic images. However, in a related study, a prototype version IMACEL was used to classify transmitted electron microscopic images of tumorigenic cancer stem cells into two categories (ABCGS2+ and ABCGS2-) [19]. However, on the IMACEL platform, we do not restrict the image acquisition tools or type of image that may be used for analysis. In fact, we are developing an extension to the image processing platform that is focused on MRI, CT, and X-ray images for specific fields of life science.

Compared with manual labelling, the classification methods in IMACEL are not highly accurate. There are several reasons for this performance in this study. First, the number of images in our dataset was small, particularly for the anaphase cell images. Deep learning is well known to perform better with a large number of images, and if one class has few examples, the resulting dataset can be imbalanced and affect the accuracy. Second, each cell cycle image was acquired using cheap fluorescent microscopy instead of a more advanced method, such as confocal laser scanning microscopy, and high levels of image noise could affect the result. Third, transfer learning could have affected the result. AlexNet was trained using not only microscopic images but also general images. Note that the above poor study conditions were selected to assess the IMACEL platform because it is aimed at researchers who do not have advanced computing skills or equipment.

A current version of the IMACEL platform, all microscopic images used in this study and detailed documentations will be distributed to interested researchers on request. Currently, we are developing three-dimensional reconstruction and the extraction of the surface area and volume for three-dimensional images. Additionally, tracking or kinetic analysis for time-sequential observations is under development.

In conclusion, we developed a new cloud-based image processing platform called IMACEL that consists of morphological analysis and image classification functions. The validation experiments indicate that particles can be extracted easily and rapidly with high accuracy. Additionally, IMACEL enables researchers to perform image classification based on machine learning without prior knowledge of image processing.

## Supporting information

S1 Fig Representative example of the manual evaluation of stress granules using ImageJ. (TIF)

S1 Movie Representative example of the IMACEL particle analyser. (MOV)

S2 Movie Representative example of morphological analysis batch processing. (MOV)

S3 Movie Representative example of the IMACEL classifier. (MOV)

S4 Movie Representative example of the output report and morphological matrix function. (MOV)

S1 Table List of the implemented image processing methods in the IMACEL particle analyser. (DOCX)
