# Supplementary figures and images for: IMACEL: A cloud-based bioimage analysis platform for morphological analysis and image classification
Source: PLoS One. 2019 Feb 22;14(2):e0212619. doi: 10.1371/journal.pone.0212619 (PMC6386377; doi:10.1371/journal.pone.0212619)

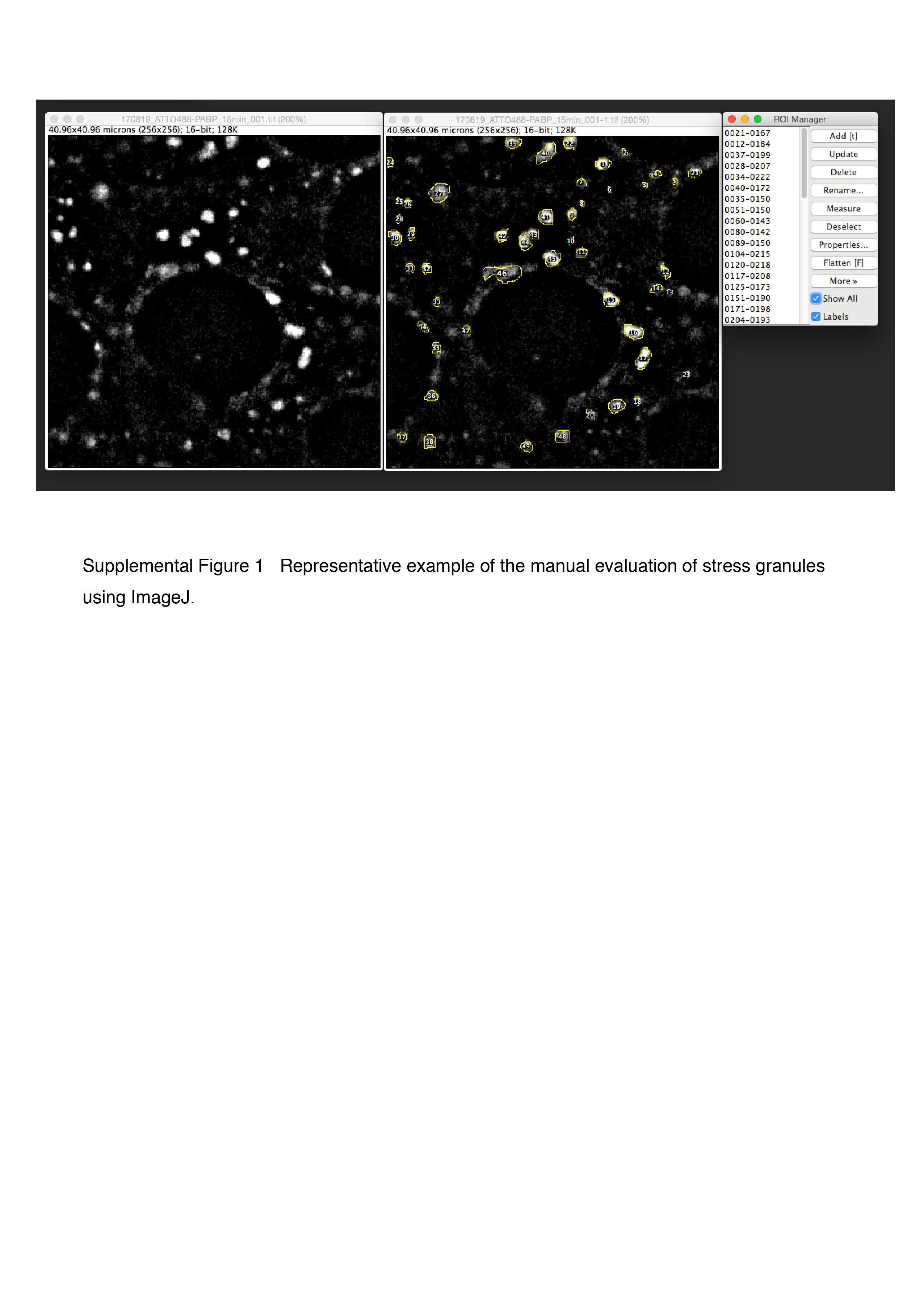

Supplement: S1 Fig — (TIF) [file pone.0212619.s001.tif]
