# Supplementary material for: IMACEL: A cloud-based bioimage analysis platform for morphological analysis and image classification
Source: PLoS One. 2019 Feb 22;14(2):e0212619. doi: 10.1371/journal.pone.0212619 (PMC6386377; doi:10.1371/journal.pone.0212619)
Supplement: S1 Table — (DOCX) [file pone.0212619.s006.docx]

Supplemental Table 1 List of the implemented image processing methods in the IMACEL particle analyser.

1. Colour channel extraction
   1. Greyscale
   2. Red channel of RGB colour
   3. Green channel of RGB colour
   4. Blue channel of RGB colour
   5. Hue channel of HSV colour
   6. Saturation of HSV colours
2. Invert to black background
3. Removing excessive bright regions
   1. Skip this operation
   2. Median filtering of bright regions (radius = 5–21 pixels)
4. Shading
   1. Skip this operation
   2. Equalization of the global intensity histogram
   3. Equalization of the adaptive intensity histogram
5. Noise reduction
   1. Skip this operation
   2. Gaussian filtering (sigma = 1 - 4 pixel)
6. Edge enhancement
   1. Skip this operation
   2. Difference of Gaussian (sigma = 0.5, 4.0 or 0.5, 8.0)
7. Binarisation
   1. Threshold = 50
   2. Otsu method
   3. Adaptive Gaussian
   4. Adaptive Mean
   5. Edge extraction using the Canny method (threshold = 100, 200 or 10, 50)
8. Noise reduction using the closing method
   1. Skip this operation
   2. Closing (radius = 3–11 pixels)
9. Fill holes
   1. Skip this operation
   2. Filling
10. Noise reduction using the opening method
    1. Skip this operation
    2. Opening (radius = 3–13 pixels)
11. Contour extraction
    1. Simple segmentation
    2. Dividing using watershed (blob ratio = 1%–15%)
